# Comparison of Azithromycin and Metronidazole in a Quadruple-Therapy Regimen for *Helicobacter pylori* Eradication in Dyspepsia

**DOI:** 10.4103/1319-3767.56091

**Published:** 2009-10

**Authors:** Shahram Agah, Babak Shazad, Babak Abbaszadeh

**Affiliations:** Department of Liver and Gastrointestinal Research, Infectious Disease Research, Iran University of Medical Sciences, Tehran, Iran; 1 Department of Tehran University of Medical Sciences, Tehran, Iran

**Keywords:** Azithromycin, *Helicobacter pylori*, metronidazole, quadruple-therapy regimens

## Abstract

**Background/Aim:**

*Helicobacter pylori* (*H pylori*) plays an important role in the pathogenesis of chronic gastritis, peptic ulcer disease, and gastric neoplasms. Therefore, it is necessary to select an effective regimen for *H pylori* eradication. The aim of this study was to compare the efficacy of two quadruple-therapy regimens—one with azithromycin and the other with metronidazole—for *H pylori* eradication in patients with dyspepsia.

**Materials and Methods:**

In this double-blind randomized clinical trial conducted in Rasoule-Akram Hospital in 2006, we included 60 patients (aged 15–70 years) who had dyspepsia and *H pylori* infection as diagnosed by upper gastrointestinal endoscopy and rapid urease test. Patients were randomly assigned to receive a quadruple-therapy regimen for 2 weeks: 1) the MAO-B group (*n*= 30) received metronidazole 500 mg b.i.d, amoxicillin 1g b.i.d, omeprazole 20 mg b.i.d, and bismuth 240 mg b.i.d and 2) the AAO-B group (*n* = 30) received azithromycin 500 mg once daily for 1 week and amoxicillin 1g b.i.d, omeprazole 20 mg b.i.d, and bismuth 240 mg b.i.d for 2 weeks). *H pylori* eradication was assessed by the rapid urease test (RUT) 2 months after the cessation of treatment.

**Results:**

*H pylori* was eradicated in 68% and 69% of patients in the MAO-B and AAO-B groups, respectively. There was no significant difference in *H pylori* eradication rates between the two groups (*P* = 0.939).

**Conclusion:**

No significant difference exists between the two quadruple-therapy regimens that were tested.

Gastritis caused by infection with *Helicobacter pylori* (*H pylori*) is one of the most common infectious diseases worldwide. *H pylori* plays an important role in the pathogenesis of chronic gastritis and peptic ulcer disease.[1–4] There is strong evidence to show that *H pylori* infection may also be associated with gastric neoplasms, i.e., carcinoma of the stomach and primary low-grade B-cell gastric lymphoma of mucosa-associated lymphoid tissue (MALT lymphoma).[5] The decrease in the incidence of *H pylori* infection in the developing countries has already led to a reduction in the incidence of gastric cancer.[6]

*H pylori* infection is one of the most common infections in Iran. In seroepidemiologic studies in different parts of this country its prevalence has been reported to be to 90% in adults older than 35 years.[7] A recent study in Ardabil in the north-western part of Iran, in which histopathology was used for screening, also revealed nearly 90% prevalence of *H pylori* infection in the normal population older than 40 years.[8]

Selection of a particular regimen for *H pylori* eradication will be influenced by several factors, including efficacy of the regimen, patient tolerance, the pattern of antibiotic resistance in the region, and the cost of the drugs. An acceptable regimen for *H pylori* eradication should show an eradication rate of 85-90% on intention-to-treat analysis.[9] Several drug regimens have been evaluated in Europe[10] and in the United States[11] for efficacy in *H pylori* eradication. Clinical experience in Iran and many other developing countries have demonstrated that the eradication rate of *H pylori* is much lower in these countries than in Western countries using the same treatment regimens; the short- and long-term recrudescence or reinfection rates are also much more than the rates reported from the West.[12]

Various regimens have been used for the eradication of this bacterium in Iran and they have shown different results. In this study, we compared the efficacy of two quadruple- therapy regimens for *H pylori* eradication in patients with dyspepsia: (A) omeprazole, amoxicillin, bismuth, and metronidazole and (B) omeprazole, amoxicillin, bismuth, and azithromycin. Regimen A is the conventional therapy in Iran.

## MATERIALS AND METHODS

### Study population

In this double-blind randomized clinical trial conducted in Rasoule-Akram Hospital in 2006, we included 60 patients (aged 15-70 years) with dyspepsia and *H pylori* infection; the cases were diagnosed by upper endoscopy and rapid urease test. Patients were not included if any one of the following conditions was present: History of antibiotic use during the 4 weeks preceding the study, gastric surgery, concurrent gastric carcinoma or other severe gastric disease, reflux esophagitis (of grade ≥2 according to Savary-Miller grading), sensitivity to any of the drugs to be used, pregnancy, or lactation. All patients had gastroenterological symptoms and were *H pylori* positive. Approval of the ethics committee of our institute was obtained before commencement of the study, as also written informed consent from all participants.

### Treatment regimens

Patients who satisfied the inclusion criteria were randomly assigned to one of two groups. The MAO-B group (*n* = 30) received metronidazole 500 mg b.i.d, amoxicillin 1g b.i.d, omeprazole 20 mg b.i.d, and bismuth 240 mg b.i.d for 2 weeks and the AAO-B group (*n* = 30) received azithromycin 500 mg once daily (for 1 week) along with amoxicillin 1g b.i.d, omeprazole 20 mg b.i.d, and bismuth 240 mg b.i.d for 2 weeks. The efficacy of the treatment was assessed by achievement of *H pylori* eradication. The urease breath test (UBT) was performed 2 months after the cessation of treatment and a negative UBT was considered to indicate eradication of *H pylori*.

### Statistical analysis

The sample size was calculated considering Chey *et al*.[13] study that found higher *H pylori* eradication rate with azithromycin 1g r/day for 3 days. The results were expressed as the mean ± standard deviation (SD) for quantitative variables andas percentages for categorical variables. Categorical variables were compared using the Chi square test; continuous variables were compared using the independent samples *t*-test for variables with normal distributions and the Mann-Whitney test for variables with non-normal distributions. *P* values of 0.05 or less were considered statistically significant. All statistical analyses were performed using SPSS version 13 and SAS version 9.1 for Windows.

## RESULTS

Patients in the two groups were matched for demographic characteristics and endoscopic findings before treatment [Tables 1 and 2]. Out of 60 *H pylori*–positive patients who entered the study, six (i.e., five patients in the MAO-B group and one patient in the AAO-B group) discontinued treatment because of the side effects of the treatment). Thus, at the end of the study, 54 patients were available for inclusion in the analysis: 27 women and 27 men, with a mean age of 42.5±10.7 years (range: 15-70 years).

**Table 1:** Patients’ characteristics in two MAO-B and AAO-B groups

Endoscopic findings	MAO-B group (*n* = 25)	AAO-B group (*n* = 29)	*P* value
Mean age (years)	42.2	42.7	0.77
Gender (n)	13 M, 12 F	14 M, 15 F	0.74
NSAID usage, n (%)	4 (16)	5 (17.2)	0.46
Smoking n (%)	6 (24)	4 (13.7)	0.14

**Table 2:** Endoscopic findings in MAO-B and AAO-B groups before treatment

Endoscopic findings	MAO-B group (*n* = 25) %	AAO-B group (*n* = 29) %	*P* value
Peptic ulcer	20.0	34.4	0.238
Antral gastritis	56.0	48.3	0.572
Body gastritis	24.0	13.8	0.336
Severe reflux	0.0	3.4	0.0013

The endoscopic findings are shown in Table 1. There were no significant differences in the incidence of peptic ulcer, antral gastritis, and body gastritis between the two groups; however, severe reflux was found to be significantly more in the AAO-B group before treatment.

*H pylori* infection was eradicated in 68% of the 25 patients in the MAO-B group and in 69% of the 29 patients in the AAO-B group. There was no significant difference in the efficacy of the two regimens for eradication of *H pylori* (*P* = 0.939).

## DISCUSSION

The prevalence of antibiotic resistance has increased substantially in recent years, and there has been a corresponding decrease in the eradication rates for *H pylori* infection.[14] Eradication rates in most Western countries have declined to unacceptable levels, with eradication therapy failing in approximately one in five patients.[15] A simple, short treatment regimen that would return eradication levels to that seen at the advent of *H pylori* treatment is urgently needed.[15] Causes of treatment failure include antibiotic resistance, poor compliance with treatment, short (7-10 days) duration of therapy, and drug-related side effects (such as skin flushing, headache, nausea, vomiting, sweating and a rapid heart rate, diarrhea, constipation, stomach cramps.).[16]

In our study, the *H pylori* eradication rate was similar in the two groups, being 68% and 69% in the MAO-B and AAO-B groups, respectively. According to published studies in Iran, about 37.5% of *H pylori* strains are resistant to metronidazole.[12] A quadruple-drug regimen, consisting of bismuth, a proton-pump inhibitor (PPI), amoxicillin, and metronidazole had good efficacy (92% eradication rate) in a study from Netherlands,[17] but this same regimen had less than 70% intention-to-treat eradication rate in Iranian studies.[18] Also, quadruple therapy (omeprazole, bismuth, amoxicillin, and metronidazole) given for 1 week was also unsuccessful.[19] This quadruple-therapy regimen consisting of omeprazole, bismuth, amoxicillin, and metronidazole has a low eradication rate and is therefore not recommended in Iran.

Azithromycin has some special attributes that suggest that it would be a promising compound to be used in regimens for *H pylori* eradication. It is acid stable, has a long half-life, and achieves remarkably high concentrations in gastric tissue.[20,21] There have been several clinical trials of azithromycin for the therapy of *H pylori* infection.[22–24]

Cammarota *et al*. found that the efficacy of clarithromycin was more than that of azithromycin; however, it had relatively more side effects than azithromycin.[25] In another study, with a regimen consisting of azithromycin, omeprazole, and metronidazole, the cure rate of *H pylori* was about 70%; however, the cure of *H pylori* infection improved gastritis.[26] Also in the Vladimir study,[27] the combination of a macrolide with amoxicillin was shown to provide higher eradication rates and therefore azithromycin at a dose of 1g per day for 3 days can be considered as a promising component of the triple PPI-based regimen.[27] In the Vladimir study, although azithromycin was administered only for 3 days, the dose was double that used by us and therefore its efficacy was similar to that in our study. Similar results were found in Chey *et al*. study.[13] In the study by Vcev *et al*., triple regimens consisting of pantoprazole, amoxicillin, and either azithromycin or clarithromycin gave poor eradication rates: 78% with the regimen containing clarithromycin and 71% with the regimen containing azithromycin.[28] According to their study, it was clear that due to the higher side effects of clarithromycin, the use of azithromycin was safer in *H pylori* eradication.

## CONCLUSION

No significant difference was detected between the two quadruple regimens that we tested for *H pylori* eradication. With the increase in the bacterial resistance to metronidazole, it seems that the use of azithromycin at a dose of 1g for 2 weeks in a quadruple regimen may be effective for achieving *H pylori* eradication.
